# Species Richness and Range Size of the Terrestrial Mammals of the World: Biological Signal within Mathematical Constraints

**DOI:** 10.1371/journal.pone.0019359

**Published:** 2011-05-06

**Authors:** Jorge Soberón, Gerardo Ceballos

**Affiliations:** 1 Biodiversity Institute and Department of Ecology and Evolutionary Biology, University of Kansas, Lawrence, Kansas, United States of America; 2 Instituto de Ecología, Universidad Nacional Autónoma de México, México D.F., México; University of Western Ontario, Canada

## Abstract

We explore global spatial diversity patterns for terrestrial mammals using as a tool range-diversity plots. These plots display simultaneously information about the number of species in localities and their spatial covariance in composition. These are highly informative, as we show by linking range-diversity plots with maps and by highlighting the correspondences between well defined regions of the plots with geographical regions or with taxonomic groups. Range-diversity plots are mathematically constrained by the lines of maximum and minimum mean covariance in species composition. We show how regions in the range-diversity plot corresponding to the line of maximum covariance correspond to large continental masses, and regions near the lower limit of the range-diversity plot correspond to archipelagos and mountain ranges. We show how curves of constant covariance correspond to nested faunas. Finally, we show that the observed distribution of the covariance range has significantly longer tails than random, with clear geographic correspondences. At the scale of our data we found that range-diversity plots reveal biodiversity patterns that cannot be replicated by null models, and correspond to conspicuous terrain features and taxonomic groupings.

## Introduction

The distribution of life on Earth has intrigued naturalists and ecologists since long time ago. Charles Darwin and Alfred R. Wallace developed their evolutionary theories partly on their insights of the patterns of distribution of plants and animals [1]. Not only studying patterns of biodiversity is important because any mechanistic explanation of species numbers and distributions would have to account for them [2], but patterns are often used to support conservation policy decisions [3].

Although it is known that contrasting mechanisms can sometimes produce the same biogeographic [4] or ecological patterns [5], it appears to be less appreciated that many measures of spatial pattern of biological diversity are strongly constrained by mathematical relations intrinsic to the definitions of species richness, beta diversity, range-sizes, dispersion-field volumes, nestedness and others [2], [6], [7], [8]. This is unfortunate, since increased availability of databases of species distributions, mostly for terrestrial vertebrates, provide an opportunity to study patterns of biodiversity at regional or global scales and resolutions ∼10^4^ km^2^. Using such databases, recent studies have analyzed, for example, patterns in species richness [9], [10], turnover [11], the shape of the species pools [12] and their ranges [13], [14], [15], occasionally attempting to display jointly measures of richness and parameters related to range of distribution [14], [15], [16]. However, limitations imposed by constraints are not yet widely discussed.

Keeping in mind the mathematical constraints existing among biodiversity indices is crucial, but interpretation of biodiversity patterns is also greatly improved by the use of software that links maps with mathematical plots. This allows exploring the relation between lines, clusters and groups of points in scatter plots and other graphs, and their geographic and taxonomic identity. Very aggregated numbers like linear correlation coefficients seldom display the wealth of structure and information present in this type of large-scale data.

We illustrate these ideas using range-diversity plots (RDP, [6]) created from presence-absence matrices (PAMs, see methods) of *S* species vs. *N* cells in a grid. The “by sites” RDP is a very informative tool, since it displays simultaneously the number of species in sites (alpha diversity) and how those species are distributed in the entire region, which has been called the “dispersion field” of Graves & Rahbek [12]. The dispersion field volume is essentially the sum of the range size of the species inhabiting a cell. A cell *i* (in a tropical island, for example) may contain many species of narrow distribution, or the opposite, a cell in the large regions of the Eurasian steppes, for instance, may have fewer but widely distributed species. These two points would appear in opposite regions within the permissible regions of a RDP plot. Christen and Soberón [17] and Arita et al. [6] recently found that several parameters related to the PAM are related by the following equation:
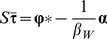
(1)Where α is a vector of the species numbers in the cells, 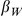 is Whittaker's [18] beta diversity, the vector 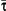 contains as elements the *N* average covariances (in species presences and absences) between each site and all the rest, and the vector 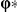 contains as elements the sum of all proportional range-sizes of the *α_i_* species inhabiting each site, which is equivalent to a vector containing the proportional dispersion field volumes of Graves and Rahbek [12]. Finally, this is equivalent to the counts of shared species that a region has with the rest of the world (see Supporting Text S2). Equation (1) implies a number of restrictions on four measures of biodiversity spatial pattern: richness, multiplicative beta diversity, mean covariance among sites, and the dispersion field volume. In particular, from (1) it follows equation (9) of Arita et al. [6]:
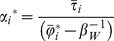
(2)which is an element by element version of equation (1) with the dispersion field of a cell divided by its number of species. Following Graves and Rahbek, [12], we call this the mean proportional range-size, or *mean range-size* to be brief: 
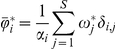
.

**Figure 1:**
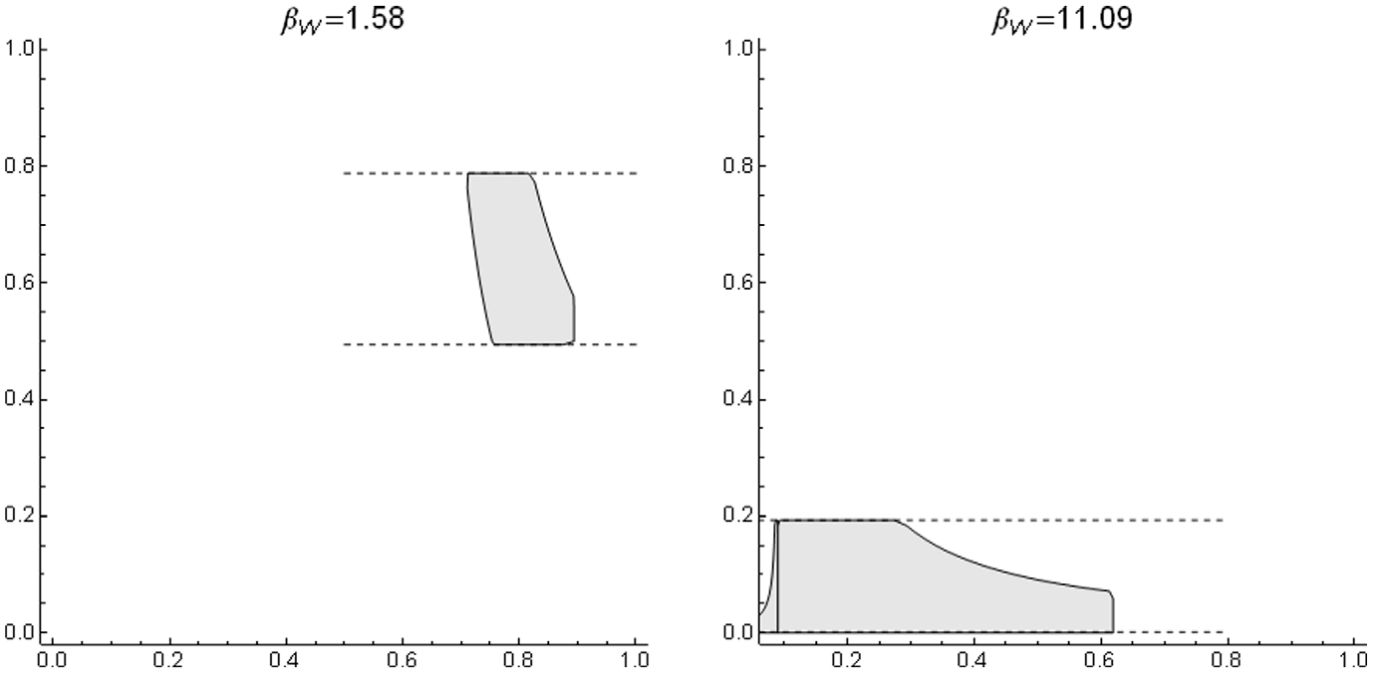
Mathematical constraints of data points plotted in a proportional richness (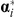) vs. proportional mean range-size (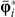) plot. The graph in the left is a relatively low beta-diversity subregion of the world (the Smoky Mountains); the graph in the right is a high beta diversity region (the entire Indo-Malay region). Both regions have cells of a resolution of 10,000 km^2^ but differing in extent (216 cells the Smokey Mountains and 951 cells the Indo-Malay region). For each one, the grey area represents the region where data points can occur, as implied by equation (2). The curved lines are the maximum and minimum isocovariance lines respectively, plotted using equation (2). The dashed lines are the maximum and minimum observed 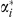.

Equation (2) imposes very strict limits to the values of several biodiversity indicators, as exemplified for two geographic regions, one of high and another of low 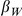 (Fig. 1). Most of the restrictions follow from the position of the extreme covariance lines and position of these, in turn, is influenced by 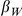 (see Supporting Texts S1 and S3). The fact that measures of richness and of range size are related to each other in ways that constrain their possible values has been, until very recently [6], consistently ignored in the literature of biodiversity patterns. In view of the above in this manuscript we apply RDPs to a database of the terrestrial mammals of the world to explore the following questions: i) what is the general shape of the RDP for the terrestrial mammals of the world? ii) Are there correspondences between different regions in the RDP and geographical regions? iii) Are there patterns in the RDP that cannot be distinguished from random factors? The emphasis we place is in the exploration of patterns.

## Methods

### Presence-absence matrices

A PAM 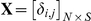 has *N* rows (cells in the grid) and *S* columns (species). This departs from the convention that places species in rows, but it is more practical when there are much more sites than species. The elements have a value 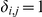 if species *j* is present in cell *i*, and zero otherwise. Local (alpha) richness is obtained from the row marginals of the matrix (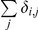), denoted by *α_i_*, and their proportional (to the total number of species) values, by α*_i_. The mean value of the richness over the entire set of rows was proposed by Whittaker [18] as a descriptor of biodiversity pattern of a region (a collection of cells). ii) Similarly, the column marginals (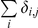) give the size of the extent of occurrence of every species [19], which, if the resolution of the grid was high, would approximate the size of the true area of distribution of the species. These are denoted by *ω_j_*, and their proportional (to the total number of cells) values by ω*_j_. Finally, iii) the total number of ones in the matrix (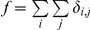) is called its “fill”, which divided by the total number of elements becomes the proportional fill: f* = f/(NS).

### Data

Our database consists of extent of occurrence [19] maps of 3,617 terrestrial mammal species, including bats. A detailed list of the references and methods used to build the database has been published elsewhere [9]. The maps are an approximation to the current extent of occurrence of these species. Extent of occurrence maps were digitized from the literature. The Earth's surface was divided into a 10,000 km^2^ per cell grid using the Behrmann equal area projection [9]. The emerged surface was represented by 16,420 cells. We defined a species range as the set of all cells that intersected the species map, creating thus a PAM of near 5×10^7^ cells. Low resolution grids are recommended when dealing with extent of occurrence maps [20] which almost never contain detailed information about the distributions of species. Moreover, increasing the resolution will make the size of a global PAM unmanageable. We subdivided the emerged surface using biogeographic regions of the world obtained from the World Wildlife Fund Ecoregions page (http://www.worldwildlife.org/science/ecoregions/item1847.html). Linkages between RDPs and maps were performed using ArcMap 9 and R scripts.

We explored patterns in the constrained space of species richness vs. mean species range of the species in a cell without attempting any statistical inference, for two reasons: 1) for the purpose of inference it is not clear to us how to deal simultaneously with the mathematical constraints in the data and its strong spatial autocorrelation. There are methods to tackle the last problem [21], [22], [23], but we are unaware of techniques to deal with constrained, autocorrelated data; and 2) in a sense, for a given resolution, a global dataset contains all the existing data about presence-absence biodiversity patterns. Hence one does not need to statistically infer rules or relationships, but merely to display them. For example, whether the Rapoport rule applies for African Chiroptera, at 10,000 km2 of resolution, does not requires extrapolation from a regression fitted for other regions or taxa: it is only a matter to check in the database. This contribution then is presented from an exploratory, rather than inferential perspective.

## Results

### Mammal Range-diversity plots

**Figure 2:**
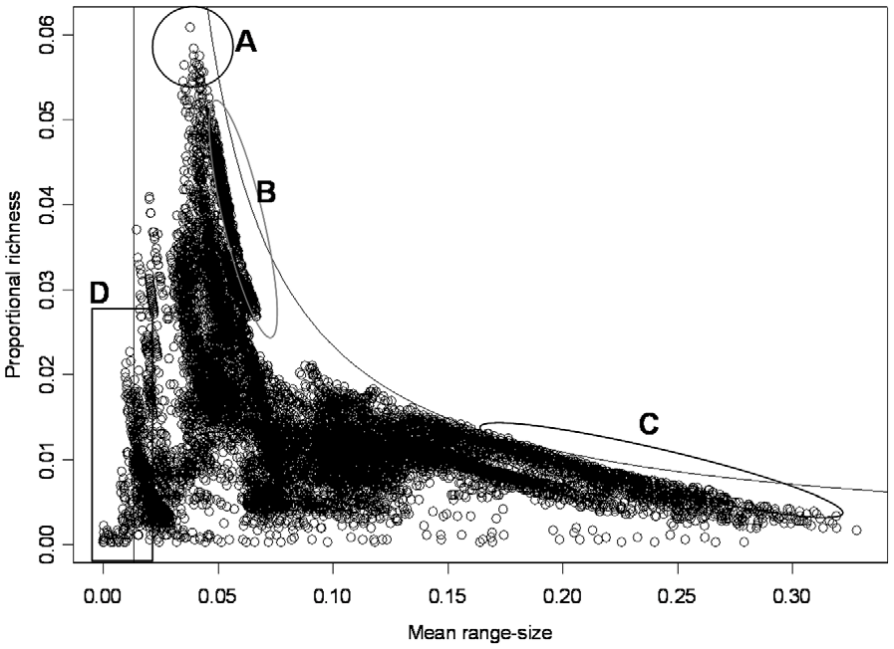
Range-diversity plot of the terrestrial mammals of the world. Data corresponds to a PAM of 16,420 cells of 10,000 km2, and 3,617 species. The vertical line is the proportional fill of the matrix, equal to 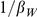 = 1/75.93. The curved line is obtained from equation (2) with maximum covariance = 2.03×10^−3^. Region (A) corresponds to the Colombian Chocó, (B) Northern South America, (C) Northern North America and Eurasia, and (D) Madagascar, the Philippines and the Malaysian Archipelago.

The RDP for all 16,420 cells in the PAM of the terrestrial mammals of the world shows substantial internal structure, with subsets of points arranged in clusters and along curved lines (Fig. 2). The value of 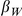 is 75.93, which means that mammalian fauna of the world is approximately 76 times larger than the average of the species numbers in all cells of 10,000 km^2^. It also means that the vertical line separating the regions of negative and positive covariances is located far to the left side of the plot, since the value of 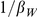 = 0.0137. This implies that the plot will have a large, “forbidden” area in the region corresponding to high richness and high mean range-sizes [6]. There are clear correspondences between geography and parts of the range-diversity plot. For example, the richest region of the world contains around 6% of the entire mammal fauna (the Colombian Chocó, Fig. 2A). The regions of the world closest to the curve of maximum isocovariance (the mathematical limit) correspond to northern South America (Fig. 2B) and Eurasia, Canada and the Northern United States (Fig. 2C). Finally, the regions with the smallest mean range sizes correspond to Madagascar, the Andes, and islands in the Philippines and Indonesian archipelagos (Fig. 2D). At the scale of the data, the different regions of the plot have straightforward geographic interpretations.

**Figure 3:**
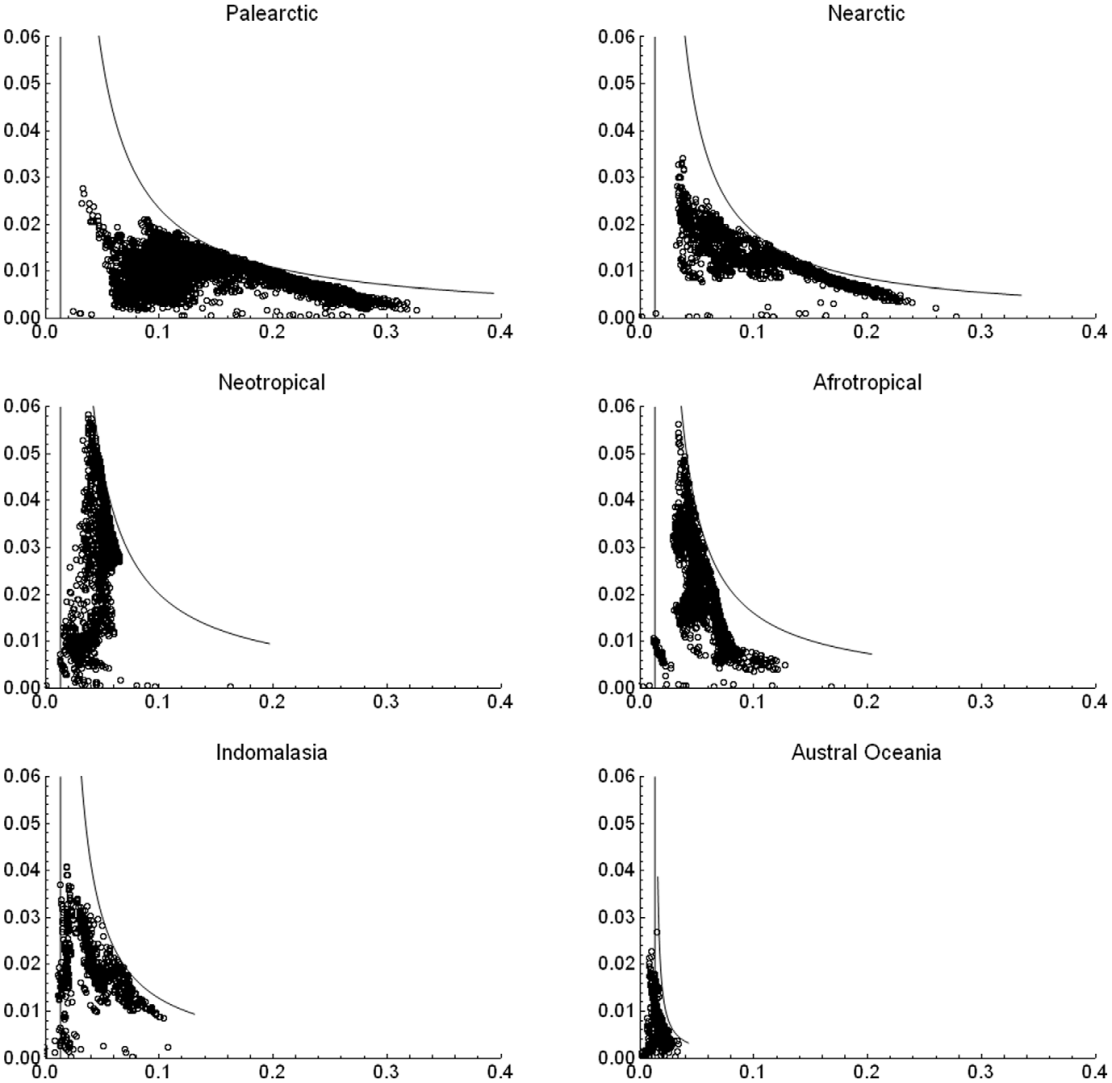
Range-diversity plot of the mammals in different biogeographical realms. The value of 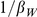 = 1/75.93 is the same for all the graphs because a single global PAM is used. Curved lines as in Figure (2) with maximum among-sites covariance: Palearctic 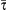 = 2.0×10^−2^, Nearctic 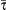 = 1.58×10^−3^, Neotropical 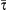 = 1.70×10^−3^, Afrotropical 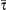 = 1.39×10^−3^, Indo-Malay 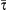 = 1.10×10^−3^, Austral-Oceania 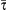 = 9.5×10^−5^.

**Figure 4:**
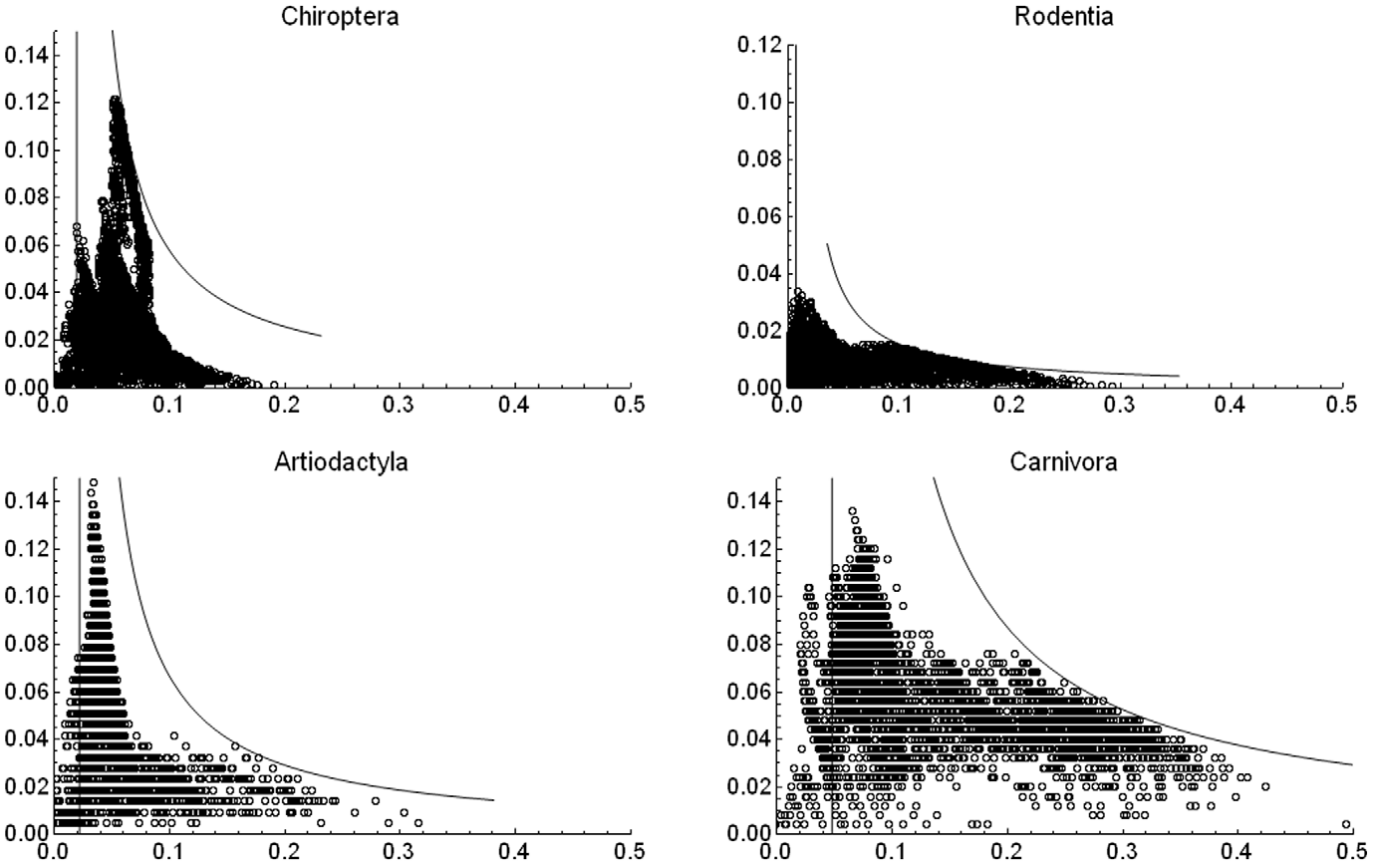
Range-diversity plot for different taxonomic groups. Boundary lines as in figure (2), with parameters: Chiroptera 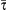 = 4.6×10^−3^, 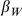 = 51.27; Rodentia 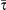
1.4×10^−3^, 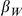 = 138.95; Artiodactyla 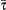 =  5.2×10^−3^, 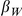 = 45.77; Carnivora 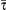 = 1.33×10^−2^, 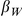 = 20.98. Beta changes because every plot was obtained from a different PAM, extracted from the global one.

It has been reported that in plots of species richness vs. mean range size “simple correlations … yield significantly negative relationships” [14], [24]. For the mammals at the scale of our analysis, generally speaking, there is indeed such a negative relation between richness and mean range (Figs. 2, 3 and 4), but this pattern is influenced to a large extent by the mathematical constraints expressed in equation (2). Rather than attempting to represent the complex pattern with a single statistic, we may ask what subregions in the plot correspond to different zoogeographic regions [25] or taxonomic groups.

### Spikes of high species numbers

**Figure 5:**
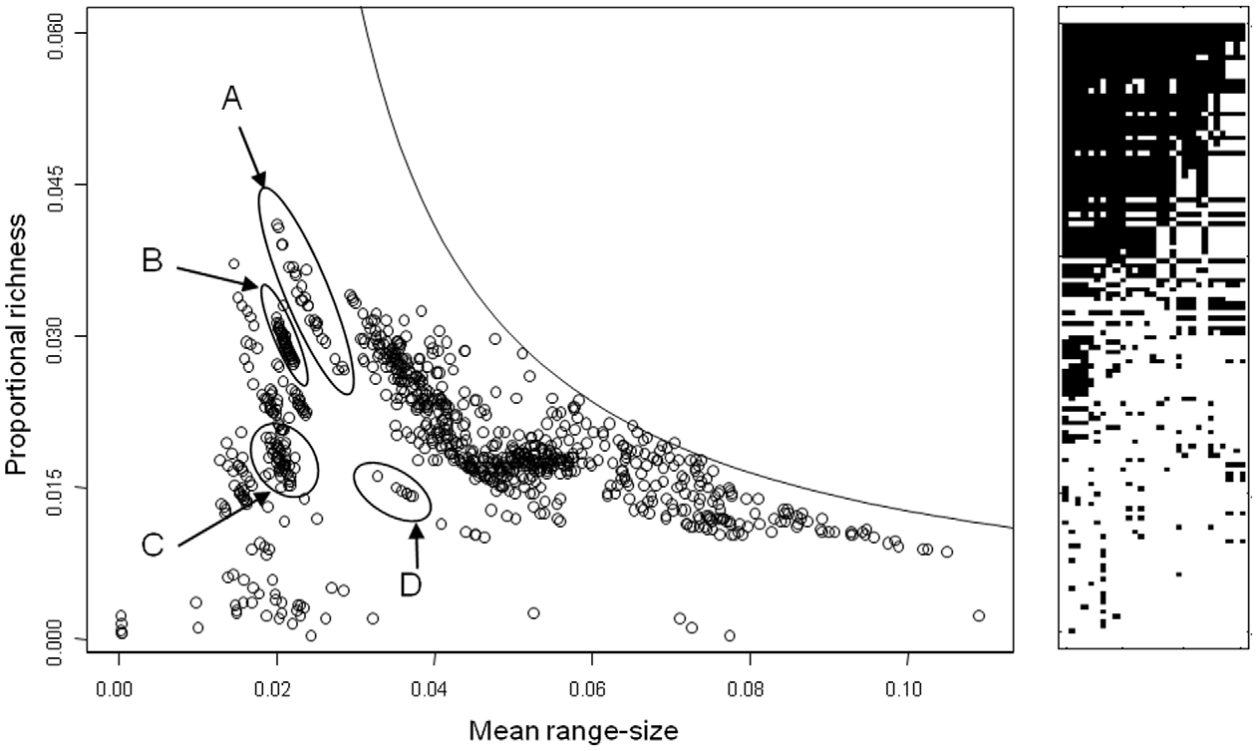
Range-diversity plot corresponding to different areas within the Indo-Malay zoogeographic region (left), and matrix plot of its PAM. The highlighted clusters in the RDP are: (A), Malay Peninsula, (B), Java, (C), Southern Borneo, (D), Sri Lanka. Line as in Figure (2) with parameters obtained from this cluster of points: τ_M_ = 1.108×10^−3^, β_W_ = 52.38. The PAM in the right is a matrix (202 species in rows and 29 cells in columns) of the Malay Peninsula cluster (A), showing its approximately nested pattern (a block of 80 species present in the entire peninsula was compacted to a single row).

The negative relation of species richness and mean range size in mammals is influenced by the fact that the maximum isocovariance line creates a narrow, funnel-like region immediately to the right of 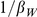, where the richest regions are located (Figs. 2, 3, 4, 5). The richest cells contain species with low to middle average range distributions, since the more species in a cell, the closest its mean proportional range size would be to the average value for all species. For the mammals of the world, the maximum isocovariance is such that regions with large numbers of species of high mean-ranges (the empty region in the graph) are mathematically impossible. In other words, at this resolution, the cells with the largest numbers of species will always have intermediate to low mean-range faunas, whereas low richness regions may have either very widespread species (large northern continental regions), or extremely narrowly spread species (small islands). A similar pattern was reported for the mammals of North America at the higher resolution of cells of ½ degrees (Arita et al. [6]. The pattern varies by region (Fig. 3) and taxa (Fig. 4), and the spike of very high proportional richness associated with relatively low mean ranges is not general.

The richest cells in the Neotropical and Afrotropical regions lay in the border of the curve of maximum covariance for those subsets, suggesting that those cells rich in species form part of contiguous large areas. Indeed this is the case, since those cells correspond to the wet forests of the northern parts of the South American continent and to the Forest-Savannas, and tropical forests ecosystems of the northern Congo, Ghana, and parts of Uganda and Kenya. On the other hand, the sets of cells near the isocovariance line in the Nearctic and the Palearctic regions correspond to low numbers of species, with very high mean ranges, corresponding to the extensive temperate or boreal continental masses of northern North America and Eurasia. The region to the left of the 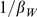 line (Fig. 2) corresponds to mountain ranges or archipelagos.

Therefore, at this scale, the RDP can be interpreted geographically, which strongly suggests that non-random mechanisms structure the regions in the plot.

### Curvilinear patterns

A second noticeable pattern is the existence of clusters and regions apparently following curved lines. What is the meaning of these structures? In the first place, we should notice that many well defined clusters correspond to geographical regions. As an example, we show some of the clusters in the Indo-Malay region (Fig. 5).

The curved sets of points correspond to lines of almost constant average covariance, or “isocovariance” lines. This is, if the average covariance 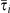 in equation (2) is equated to a particular value (in practice, a small interval) within its observed range, plotting displays hyperbolic lines with the cells having values in that interval of average isocovariance following the line [6], [17]. Isocovariance then acts like a filter that extracts subsets of the matrix, and at this global scale and low resolution, contiguous points along the isocovariance lines tend to belong to natural geographic regions, (Fig. 5). The cluster of points in the southern part of the Malaysian Peninsula corresponds to the narrow covariance interval 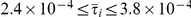. Given the global extent of the dataset, cells that are geographically unrelated may have the same mean isocovariance. For example, at a global extent, the inequality above extracts, besides the Malaysian peninsula, regions in South Africa, the Arabian Peninsula, Northern Argentina and Baja California (not shown). It is very unlikely that these regions have much in common, besides the fact that the values of 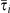 lie within the same narrow range. Therefore, interpretation should be subject to identifying geographically sensible sets of cells.

Arita et al. [6] showed that perfectly nested communities (i.e., with a triangular PAM) would appear in a range-diversity plot as straight lines with a slope of −2. This suggests that subsets of cells selected by the isocovariance filter may also have some sort of regular structure in the corresponding sub-PAM. Indeed this is the case, as shown in a matrix-plot (Fig. 5). The PAM extracted by the isocovariance filter in (Fig. 5) shows an apparently non-random pattern. The isocovariance filter not only has geographic meaning, but also appears to identify a nested PAM. Given the current debate on how to properly measure nestedness in PAMs [26], [27], [28], in this contribution we limit ourselves to notice that isocovariance filter seem to extract partially nested matrices. In another work [17] we show how the isocovariance filter allows extraction of nested matrices embedded in completely randomized PAMs.

### Distribution of mean covariances

**Figure 6:**
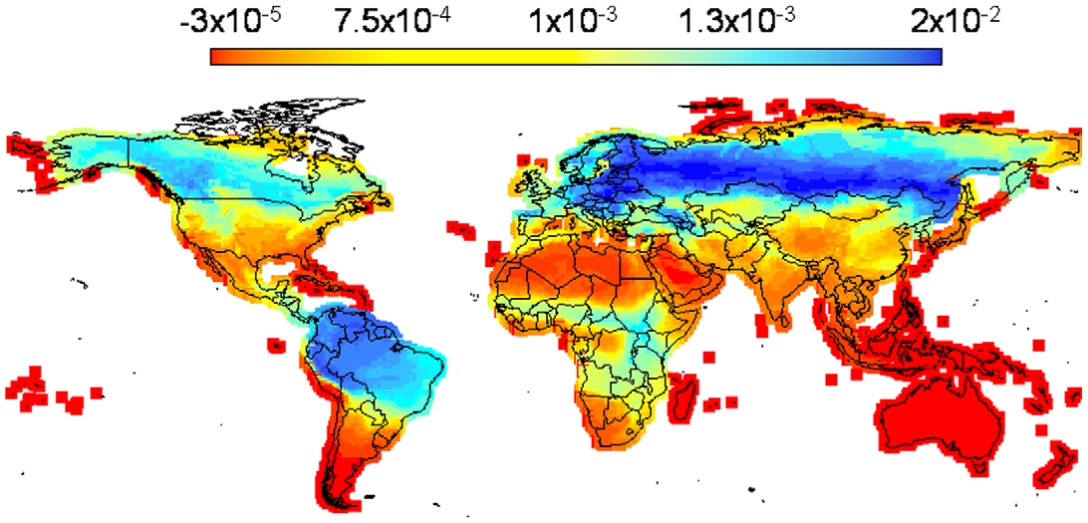
Geographic distribution of the mean covariance for the terrestrial mammals of the world. Notice how the lowest values of covariance (red) tend to occur in islands, mountain ranges and the periphery of continents, while the largest covariances occur in the bulk of continental regions.

We have seen that the mean covariance of the species composition of cells is an important number determining the shape and size of the cloud of points in a range-diversity plot, and with the potential to be used as “filter” to extract geographically and ecologically meaningful subsets. The covariance of binary ranges of two species over all sites is a measure of co-distribution of species which has been used to analyze biodiversity patterns at a community scale [5]. The corresponding analysis by sites is the covariance of community composition (over all species) between pairs of sites. The average over all sites is the measure of mean covariance in equation (2). A map of mean covariance shows the regions most dissimilar/similar, on average, to the rest of the world (Fig. 6).

**Figure 7:**
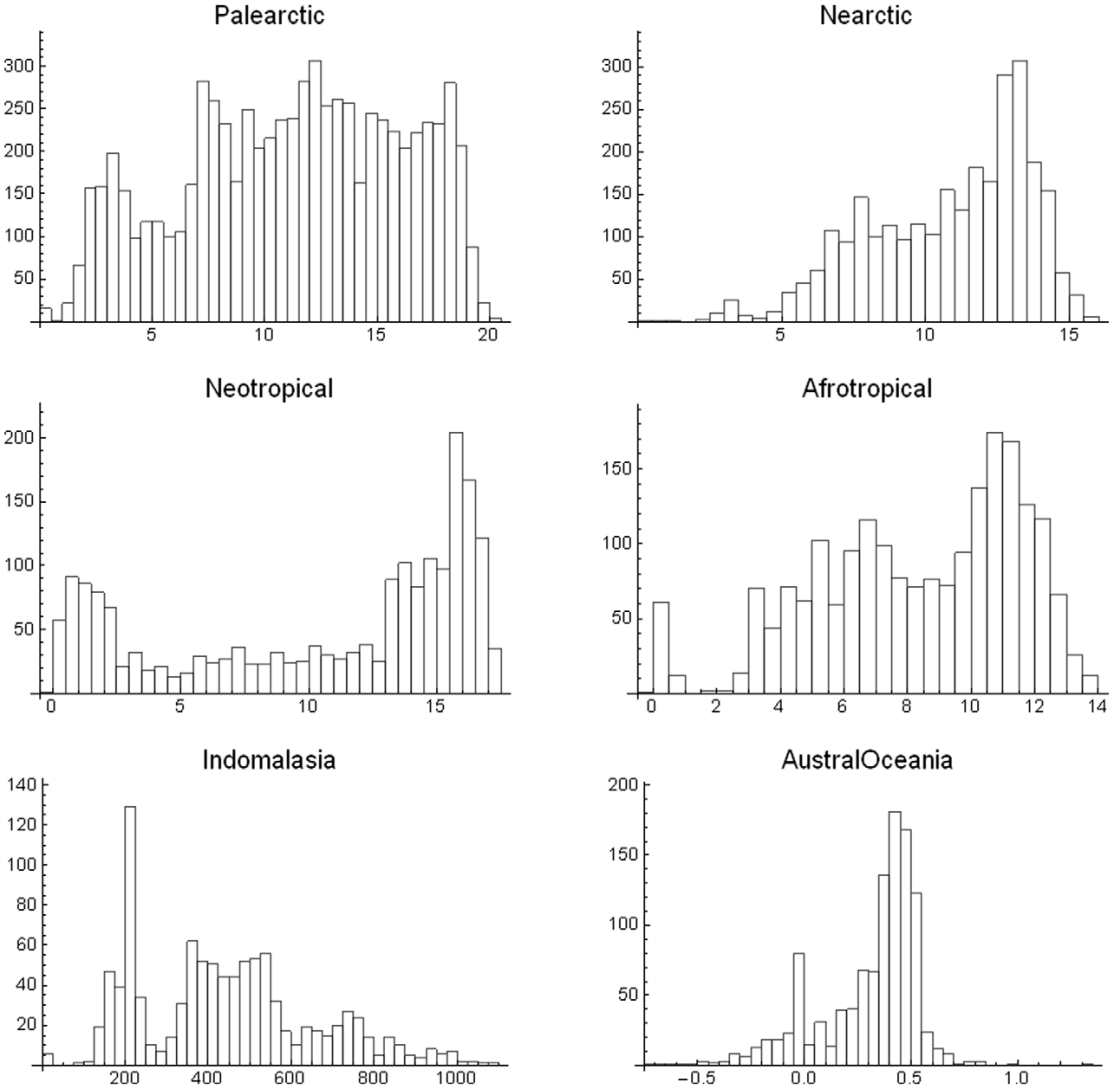
Histograms of mean covariances ×10^4^ for cells in different biogeographic realms.

The covariance distributions change with zoogeographic region or taxonomy. One would expect that ecological and evolutionary non-random processes would produce long (in relation to random models) positive and negative tails in such distributions, due to adaptation to local conditions, high spatial covariance of certain environments, and interactions with other species [5]. Histograms of the distributions of the mean covariance for the six main zoogeographic regions of the world (Fig. 7) suggest that all the distributions are multimodal to different extents. The geographical interpretation of the tails and some modes is illuminating. For instance, the rightmost cluster of bars in the Nearctic region corresponds to the northern part of the continent, in northern Canada (regions with low numbers of species very dissimilar, on average, from the others), and the left tail corresponds to the south-pointing peninsulas of North America. The leftmost group of bars in the African region corresponds to the island of Madagascar (i.e. with many geographically restricted species), and its right tail to the central part of sub-Saharan Africa. The leftmost cluster of bars in the Neotropical region corresponds to the Southern tip of the continent, and to the Pacific side of the Andes, and the cluster of bars to the right correspond mostly to the tropical wet part of Northern South America. The left side part of the distribution in the Indo-Malay region corresponds almost exactly with Sri Lanka and the archipelagos of Indonesia and the Philippines (the right part of the bimodal distribution includes almost all the continental part of the region). Finally, the leftmost cluster of bars in the Austral-Oceania region corresponds to the islands, whereas the right cluster corresponds almost entirely to the bulk of the Australian continent. So, the mean covariance histogram appears to capture a very clear geographic signature from the data: low mean covariances are related to regions in the peripheries of the continents, archipelagos and mountain ranges, and high mean covariances to the bulk or the central parts of them. Fig. S1 in the Supporting Information illustrate these ideas.

**Figure 8:**
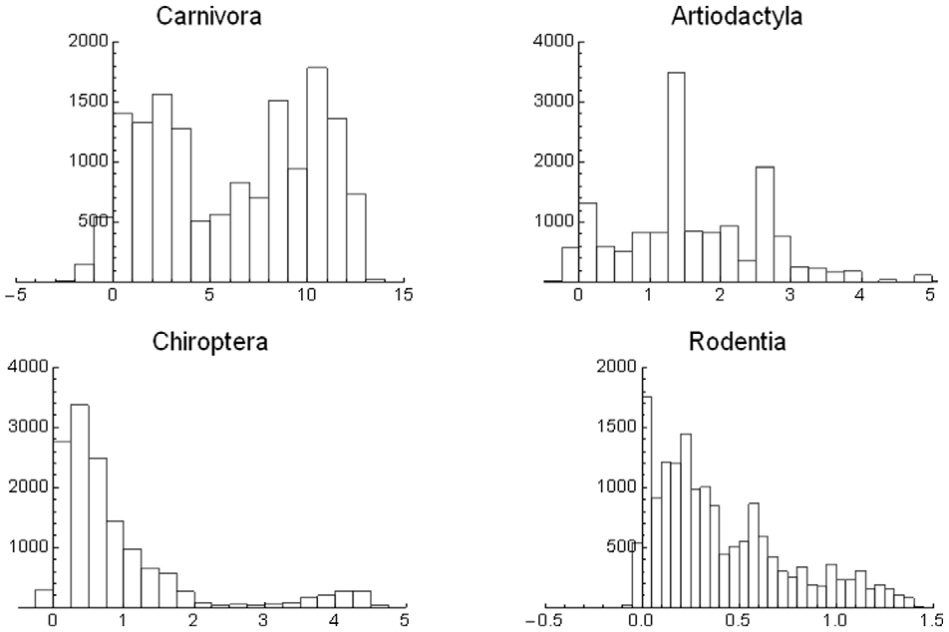
Histograms of mean covariance ×10^4^ of cells in the world, for different Orders of mammals.

How is the mean covariance distributed when disaggregated taxonomically? In Fig. 8 this question is explored. For the Carnivora there is a clear bimodal distribution. The bars in the leftmost distribution correspond to most of the tropical part of the world with species with relatively small geographic ranges, and the right mode and bars around it to large regions in the temperate north, with species with very large geographic ranges. In other words, there appears to be a large number of cells with species of large mean range. The Artiodactyla display a different pattern; there is neither clear bimodality nor simple geographical relations. The central high-percentage bar includes cells in northeastern South America, parts of Canada and Alaska, and isolated cells in Africa, Central Asia, and Southeast Asia. The Chiroptera present a strikingly regular–looking bimodality. The categories with high mean covariance correspond entirely to the northern part of South America, including most of Brazil, the Guyanas and Venezuela and Colombia. In other words, the set of cells with the highest covariances are all located roughly in the same region of tropical America but the left part of low mean covariance corresponds to areas all over the world. Finally, the mean covariance for the rodent species shows a very left-skewed distribution, with a very large peak of frequency at the smallest mean size.

Using different biogeographic regions and taxonomic groupings change drastically the distribution of mean covariances and the range-diversity plot, and those differences may be interpreted in terms of dispersal abilities, geographical shape and the extent of dominant biomes. For example, carnivores, that have probably larger home ranges and dispersal abilities than most rodents [29] produce communities with consistently larger mean covariances, and the bats, also with large dispersal capacities, present a peak of large mean covariance. This is in large measure due to the range-size distributions which for these groups are right skewed. Geographically, most archipelagos and regions of the world with north-south mountain ranges are associated with low covariances, and large continental masses are associated with high mean covariances.

### Null models and the range-diversity plot

The patterns we have displayed up to this point can be consistently explained by considerations about shapes of continents and very coarse assumptions about the mobility capacities of different taxa. How different are the patterns from what would be obtained from different classes of null and neutral models [5], [30]? One way to answer this question is by randomizing the PAMs subject to different constraints. For null models, randomizing large PAMs when both the marginals are kept constant is not a simple problem [31], [32]. In fact, by using available software like EcoSim [31], we are limited to matrices of at most 300×800 elements, or about 0.4% of the total size of the PAM we use here.

The problems of attempting a neutral model for the mammals of the world with a realistic geography are much more daunting, since a number of essentially arbitrary decisions need to be taken about dispersal and speciation rates, and software capable of tackling realistically big regions is not available. In the near future general simulation models (GSM) will become available [33] and null modeling of realistic, large-scale communities may become feasible.

**Figure 9:**
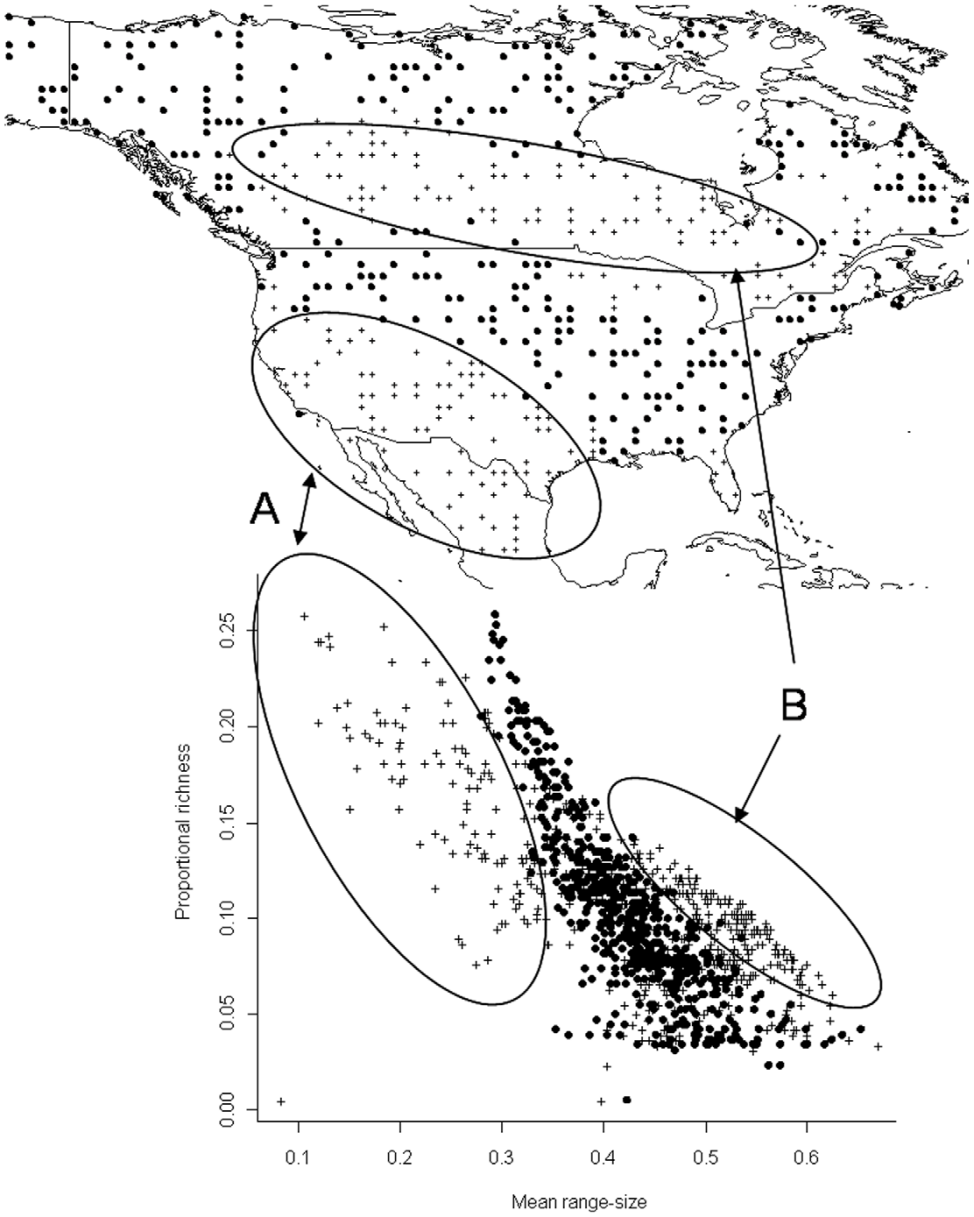
Range-diversity plots for the observed (plus signs) and one constrained randomization of a 600×379 PAM (closed circles). The ellipses A and B, which approximately correspond to geographical regions, have low or no overlap with the randomization results. Points in A (with abscissa value <.30) have relatively narrow mean ranges and higher species richness than most of the rest. Points in B contain the largest mean ranges. See text for the discussion of the geographic counterpart.

**Figure 10:**
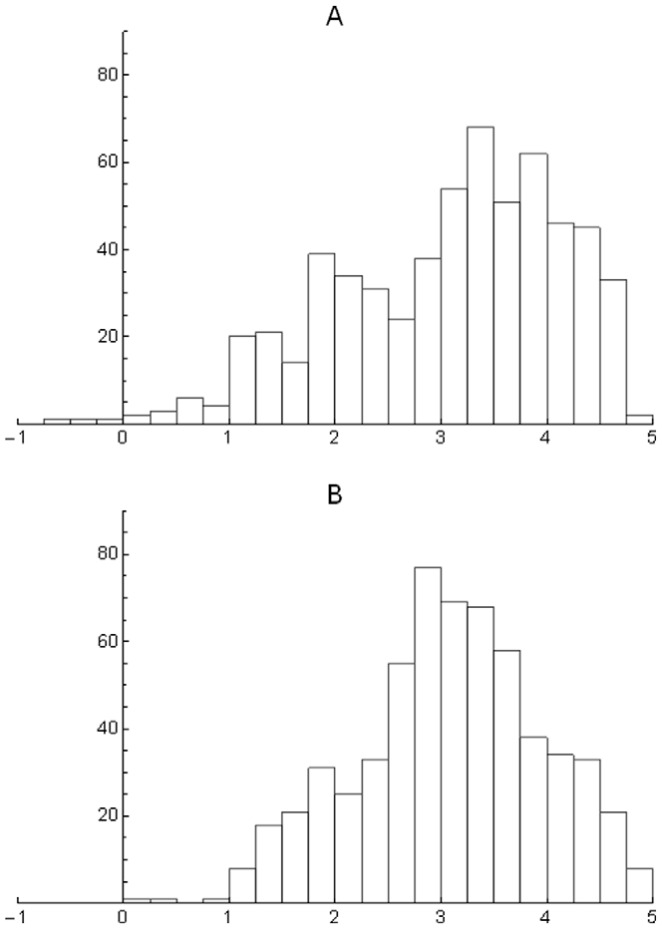
Distribution of mean covariances in the data (A), and in one instance of the null model (B).

We explore a simple null model, and for the purpose of illustration, we selected a random subset of 600 cells in the Nearctic region (the random subselection probably affect the autocovariance structure of the data, but the appearance of the RDP remains unchanged). This subset contains 379 species and the size of its PAM allows use of EcoSim. The PAM was randomized subject to fixed marginal values [32]. In other words, only randomizations of the cell values that maintained the observed distributions of local richnesses and species distribution sizes were used. EcoSim produced 100 such constrained random matrices that we used as null models. As an example we present a RDP of one of the constrained randomizations together with the observed data (Fig. 9). The richness values are the same in both datasets, but abscises may differ. The chosen randomized matrix appears to have a narrower distribution of mean range-size than the observed one. This is equivalent to say (see equation 2) that the sample of the Nearctic data has a broader distribution of covariances than the constrained null model. Indeed this is the general case. In Fig. 10 we present a histogram of the covariance values for the data and for one instance of the 100 null models. Fig. 10 shows that the data has a longer tail towards the smaller values of 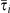 and an accumulation of higher frequencies towards the largest value of 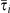. The two distributions differ significantly (Kolmogorov-Smirnoff D = 0.105, p = 0.00268). A histogram of the range of 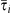 in the 100 simulations shows that the observed range is found completely outside the distribution for the null models (Fig. S2 in the Supporting Information).

What is the meaning of these results? As before, geographical interpretation of the range-diversity plot is illuminating. Constrained randomization produces regions in the range-diversity plot that overlap with a substantial number of observed points (Fig. 9). However, the observed data contains a large number of points outside the random cloud. First, there is a group with small covariances which occur in the southern part of the Nearctic region (region A in Fig. 9). Cells in region A contain relatively larger numbers of species, but their similarity to other regions is low. Remember that the mean range size field is the proportion of shared species. Therefore region A, one of the two that do not overlap with constrained randomization, contains cells with few shared species elsewhere in the region, but with a broad range of possible richness values. The other non-random region occurs in a band along northern USA and southern Canada (region B in Fig. 9). This is a region of high covariance (or high proportion of shared species) and low or intermediate number of species. Further north, regions have mean range-sizes larger but richness is much smaller. The data maintains a clear non-random signal associated to the tails of the covariance distribution. The EcoSim algorithm used in this null model therefore cannot produce these extreme situations, which suggest that historical or geographical factors may be needed to explain these parts of the biodiversity pattern. Similar results were recently obtained by Borregaard and Rahbek [34] and Villalobos and Arita [35] using null models based on a spreading-dye algorithm with constant range-sizes. They found that the spreading-dye null model overlaps mostly with the central part of the RDP, but the periphery of the observed pattern does not overlap with the simulation.

## Discussion

Spatial patterns of biodiversity are often summarized using a bewildering variety of indices. By resorting to the recently uncovered mathematical relationship between several such indices the analysis of pattern gains in rigor and depth. The constraints equations precisely describe the relation between several diversity measures, but it tells little about the statistical properties of the numbers related by it. It would be possible, even desirable, from a statistical point of view, to use some other indices of the concepts related by the equation. For instance, given that range distributions may be skewed, the median of the dispersion field volume may be statistically more appealing than its average (see [36] for a method to remove the effect of skewed distributions of range size). However without an equation expressing precisely the relation of such an index to other concepts we are back to the situation where constraints are unknown.

We found that RDPs and mean covariances are informative descriptors of patterns. The maximum and minimum values of the vector of mean covariances 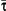 determine the available region in a range-diversity plot, and their observed distributions differ significantly from null-model covariance distributions. For the mammals of the world, at the scale we used, we showed that within the permitted region in the plot there is a very substantial amount of structure in the data, and the different regions of the graph often have straightforward geographic (i.e., non-random) correspondences. Clear geographic signals are the norm when analyzing global patterns of biodiversity [14], [15], [37] but the use of suitable software to explore the patterns by linking the abstract plots with maps provides further insights. The constant covariance lines “extract” highly non-random subsets from the apparently disorganized cloud of points. Specifically, points near the highest covariance line correspond to large, contiguous regions of the world, with many species in the tropics, and fewer in the north, and/or to large or highly mobile taxonomic groups. Regions near the minimum covariance line are clearly associated with fragmented geographies, like archipelagos or mountain ranges, or the tips of continents.

It would be possible to predict that other vertebrates may present the same general pattern, but since at a given scale of analysis, multiplicative beta diversity should be larger for groups like amphibians and reptiles and smaller for birds [38], then the permitted regions will differ, and the histograms of mean covariances should be biased towards lower values in reptiles and amphibians, and the reverse in birds. McKnight et al. [37] have shown that areas of high “beta diversity” (specifically, a measure of rate of change in composition similarity as a function of distance), coincides geographically for birds, mammals and amphibians and reptiles. Mean-range is a measure of compositional similarity (see Supporting Text S2), and therefore we would also expect low mean-ranges to coincide geographically for birds, mammals and amphibians.

Null models obtained by randomizing the original PAM subject to fixed marginal values yield range-diversity plots with a substantial, but incomplete overlap with the data. In the example we used for the null model, the regions of observed points that do not overlap with the randomized case correspond to the extreme-valued covariances (roughly extreme mean range-sizes) which are associated either with the periphery of the region (small covariances) or with the bulk of the continental extent (high covariances). In an ecological context, the reason for high covariances may be due to the existence of suites of species adapted to extensive and spatially autocorrelated environments [5]. Conversely, small covariances should correspond to regions containing species in the border of the available land, or inhabiting very unique environments. This pattern appears to be very general for the global terrestrial mammals database we used and has clear implications for conservation strategies, since these low covariance regions are the most unique and unrelated in terms of species composition, but seldom appear to present the largest species richness that are so attractive to policy makers [9], [39].

Our results indicate very clearly that at the global scale and low resolution of our study, an entire taxon displays a large number of patterns that are very different from those created by a strongly restricted null-model. Moreover, the use of the RDP method, and specifically the exploration of subregions defined by constant and/or extreme covariances reveal structures that strongly suggest that geography, history, and the dispersal capacities of mammals may leave a clearly detectable signal within the mathematically permitted region of the range-diversity plot. Proving that a given pattern is actually caused by non-random factors is a daunting task, but the range-diversity plot is a very helpful tool to identify and isolate regions of the world, or sub-taxa that are the best candidates for such non-random explanations.

## Supporting Information

Text S1 Derivation of equations 1 and 2.
(DOC)

Text S2 Proof that the mean range equals the proportion of species shared.
(DOC)

Text S3 Interpretation of Main Text Figure 1.
(DOC)

Figure S1 Extreme covariances and corresponding geographic regions for the Palearctic and Afro Tropical Regions. Histogram of covariances ×10^4^ (right column) and corresponding regions in the map, displaying the fact that extreme values of covariance are associated to large, contiguous regions of similar ecological conditions (high covariance, purple) or to islands or peripheral regions of regions (low covariance, orange).
(TIF)

Figure S2 A histogram of the range of values for the range in mean covariance in 100 EcoSim randomizations (A), and in the observed Nearctic PAM (B).
(TIF)
