# Supplementary material for: Species Richness and Range Size of the Terrestrial Mammals of the World: Biological Signal within Mathematical Constraints
Source: PLoS One. 2011 May 6;6(5):e19359. doi: 10.1371/journal.pone.0019359 (PMC3089617; doi:10.1371/journal.pone.0019359)
Supplement: Text S1 — Derivation of equations 1 and 2. (DOC) [file pone.0019359.s001.doc]

**Supporting Information 1. Derivation of equations 1 and 2**

Here we provide an alternative explanation to materials found in (1) and (2) and that explains the constraints equation. A first constraints equation was proposed by Whittaker (3), when defining his first measure of beta diversity:

$\beta_{W}\equiv N/\bar{\varpi}=S/\bar{\alpha}$ . . . (SI1)

Although Whittaker proposed equation (SI1) as a definition of what later become to be known as the multiplicative measure of beta diversity, by the definitions given above:

$N\bar{\alpha}=\sum_{i=1}^{N} \alpha_{i}=f=\sum_{j=1}^{S} \omega_{j}=S\bar{\omega}$. . . (SI2)

From which it follows immediately that$\bar{\alpha}^{*}= \bar{\alpha}/S=\bar{\omega}/N=\bar{\omega}^{*}$. The quantity *f* is called the “filling” of the matrix. From this equation, the multiplicative beta diversity measure is simply the reciprocal of the proportional average range size of the species: $\beta_{W}^{-1}=\bar{\omega}/N=\omega^{*}$ (1, 4-6), and notice that the above also means that $\beta_{W}^{-1}=f*=f/NS$. The quantity$\beta_{W}$ is the simplest and most used measure of beta diversity (7). Besides being the reciprocal of the mean range size of the species that inhabit a region, $\beta_{W}$ is the factor relating the total number of species in a region, *S,* to the average of local richness, $\bar{\alpha}$.

Equation (SI1) relates the most elementary measures of pattern that one can study. Unfortunately, these very popular measures of pattern capture only very general features of the PAM: indeed, as follows immediately from equation (SI2), they are invariant to any transformation of the PAM that do not affect its dimensions and fill (1, 8). An example of such transformation is fragmenting the distributions and shuffling the fragments. As long as the number of species, the size of the region, and the total area of distribution of each species remains constant, the values *f*, *N* and *S* and thus of $\bar{\alpha}$ and $\beta_{W}$ will also remain constant. In other words, $\bar{\alpha}$ and $\beta_{W}$ do not capture important features of biodiversity pattern, and this immediately suggests the need for more sensitive measures (1, 9).

A natural next step is to use the covariance between the communities in two sites [or, respectively, the covariance between areas of distribution. See Arita et al. (1)]. The covariance between cells *i* and *h* is simply$c(i,h)=\frac{1}{S}\sum_{j=1}^{S} \delta_{i,j}\delta_{h,j}-\frac{\alpha_{i}}{S}\frac{\alpha_{h}}{S}$. The average covariance between sites *i* and all the rest is denoted as$\bar{\tau}_{i}=\frac{1}{N}\sum_{h=1}^{N} c(i,h)$.

We define the total range-size of the species living in site (or cell) *i* to be$\phi_{i}=\sum_{j=1}^{S} \delta_{i,j}\omega_{j}$. This is the “dispersion field volume” of Graves and Gotelli (10). The mean range-size of a site is then$\bar{\phi}_{i}=\frac{1}{\alpha_{i}}\sum_{j=1}^{S} \delta_{i,j}\omega_{j}$, and the corresponding proportional mean range-size$\bar{\phi}*_{i}=\bar{\phi}_{i}/N$. Consider now the presence-absence matrix

$\mathbf{X}=\left[\begin{matrix} \delta_{1,1} & \cdots& \delta_{1,S} \\ \vdots& \ddots& \vdots\\ \delta_{N,1} & \ldots& \delta_{N,S} \end{matrix}\right]$, and its correspondent row-averages matrix (remembering that the asterisk denotes proportional values):

$\bar{\mathbf{X}}=\left[\begin{matrix} \frac{1}{S}\sum_{j=1}^{S} \delta_{1,j} & \cdots& \frac{1}{S}\sum_{j=1}^{S} \delta_{1,j} \\ \vdots& \ddots& \vdots\\ \frac{1}{S}\sum_{j=1}^{S} \delta_{N,j} & \ldots& \frac{1}{S}\sum_{j=1}^{S} \delta_{N,j} \end{matrix}\right]=\left[\begin{matrix} \alpha_{1}^{*} & \cdots& \alpha_{1}^{*} \\ \vdots& \ddots& \vdots\\ \alpha_{N}^{*} & \ldots& \alpha_{N}^{*} \end{matrix}\right]_{N\times S}$.

By definition, the variance-covariance matrix of the presences in the sites is $\mathbf{\Sigma}=\frac{1}{S}(\mathbf{X}-\bar{\mathbf{X}})(\mathbf{X}^{T}-\bar{\mathbf{X}}^{T})=\frac{1}{S}(\mathbf{XX}^{T}-\bar{\mathbf{X}}\bar{\mathbf{X}}^{T})$

And the average covariance between a site and all the others (including it) is:

$$\bar{\mathbf{\tau}}=\frac{1}{N}\mathbf{\Sigma1}_{N,1}$$

where the vector $\bar{\mathbf{\tau}}$ contains the average values of the covariances and **1**_N,1_ is a column vector of ones. Therefore,

$$SN\bar{\mathbf{\tau}}=S\mathbf{\Sigma1}=(\mathbf{XX}^{T})\mathbf{1}-(\bar{\mathbf{X}}\bar{\mathbf{X}}^{T})\mathbf{1}=\mathbf{X}(\mathbf{X}^{T}\mathbf{1})-(\bar{\mathbf{X}}\bar{\mathbf{X}}^{T})\mathbf{1}$$

But $\mathbf{X}(\mathbf{X}^{T}\mathbf{1}) =\mathbf{X}\left[\begin{matrix} \sum_{i=1}^{N} \delta_{i,1} \\ \vdots\\ \sum_{i=1}^{N} \delta_{i,S} \end{matrix}\right]=\mathbf{X}\left[\begin{matrix} \omega_{1} \\ \vdots\\ \omega_{S} \end{matrix}\right]=\left[\begin{matrix} \sum_{j=1}^{S} \delta_{1,j}\omega_{j} \\ \vdots\\ \sum_{j=1}^{S} \delta_{N,j}\omega_{j} \end{matrix}\right]=\left[\begin{matrix} \phi_{1} \\ \vdots\\ \phi_{N} \end{matrix}\right]$, and

$(\bar{\mathbf{X}}\bar{\mathbf{X}}^{T})\mathbf{1}=\frac{1}{S}\left[\begin{matrix} \alpha_{1}^{2} & \cdots& \alpha_{1}\alpha_{N} \\ \vdots& \ddots& \vdots\\ \alpha_{N}\alpha_{1} & \cdots& \alpha_{N}^{2} \end{matrix}\right]\mathbf{1}=\frac{1}{S}\left[\begin{matrix} \alpha_{1}\sum_{i=1}^{N} \alpha_{i} \\ \vdots\\ \alpha_{N}\sum_{i=1}^{N} \alpha_{i} \end{matrix}\right]=\frac{f}{S}\left[\begin{matrix} \alpha_{1} \\ \vdots\\ \alpha_{N} \end{matrix}\right]$, where the total number of ones is *f*, the filling of the matrix **X**. As we saw, by definition *β_W_* = *NS/f* Therefore we obtain:

This is equation (1) in the text, and element by element it can be expressed as:

$$\begin{matrix} S\bar{\tau}_{i}=\alpha_{i}\left(\bar{\phi}_{i}*-1/\beta_{W}\right) \\ \alpha_{i}*=\frac{\bar{\tau}_{i}}{\left(\bar{\phi}_{i}*-1/\beta_{W}\right)} \end{matrix}$$
