# Supplementary material for: Species Richness and Range Size of the Terrestrial Mammals of the World: Biological Signal within Mathematical Constraints
Source: PLoS One. 2011 May 6;6(5):e19359. doi: 10.1371/journal.pone.0019359 (PMC3089617; doi:10.1371/journal.pone.0019359)
Supplement: Text S2 — Proof that the mean range equals the proportion of species shared. (DOC) [file pone.0019359.s002.doc]

**Supporting Information 2. Proof that the mean range equals the proportion of species shared.**

The *N*x*N* matrix $\mathbf{A}=\mathbf{XX}^{T}$ contains in its diagonal, the richness of sites *1,2,…N*, denoted by *α_i,i_* and in the off-diagonal elements, the number of species shared by two sites, and denoted by *α_i,h_*. This follows from the fact that element *α_i,h_* in the **A** matrix is simply $\alpha_{i,h}=\sum_{j=1}^{S} \delta_{i,j}\delta_{h,j}$, which, since the delta values are 1 or 0, is but a count of all the joint occurrences of all *S* species in localities *i* and *h*.

The matrix $\Omega=\mathbf{X}^{T}\mathbf{X}$ of dimension *S*x*S*, contains the range value of each species, along the diagonal, and the number of localities shared by species *j* and *k*, *ω_j,k_*, off the diagonal.

Let ***ω*** = the vector of values of ranges of the *S* species, and recall that **ϕ** = the vector of the total values of the ranges of the species inhabiting the *N* sites. Since $\omega_{j}=\sum_{i=1}^{N} \delta_{i,j}$ then it is obvious that$\mathbf{\omega}=\mathbf{1X}$, where **1** is a row vector of *N* 1s. And since$\phi_{i}=\sum_{j=1}^{S} \delta_{i,j}\omega_{j}$, then it is also obvious that:

$$\begin{matrix} \phi= \mathbf{\omega X}^{T}=(\mathbf{1X})\mathbf{X}^{T}=\mathbf{1}(\mathbf{XX}^{T}) \\ =\mathbf{1A} \end{matrix}$$

In other words, the sum of the ranges of a species in a site equals the sum of the shared species of that site with all the other sites in the world. The mean-range vector is then:

,

which, by the preceding equation, is the vector of total shared species of each site, proportional to the richness of each site. The vector of mean proportional ranges $\bar{\phi}*$ is therefore equivalent to a vector of average proportional (to its richness) shared species of every site to the rest.
