# Supplementary material for: Species Richness and Range Size of the Terrestrial Mammals of the World: Biological Signal within Mathematical Constraints
Source: PLoS One. 2011 May 6;6(5):e19359. doi: 10.1371/journal.pone.0019359 (PMC3089617; doi:10.1371/journal.pone.0019359)
Supplement: Text S3 — Interpretation of Main Text Figure 1. (DOC) [file pone.0019359.s003.doc]

**3. Interpretation of Main Text Figure 1.**

The vertical line, which crosses the abscises at a value of $\beta_{W}^{-1}$, separates the graph in regions of positive and negative average covariances, respectively. The curved lines are obtained by substituting the maximum (and minimum) observed average covariances in equation (2) and plotting for an interval of the observed values of. The maximum and minimum covariance lines provide the best limits to the scatter plot of $\alpha_{i}^{*}$ vs. and are an improvement over the approximate limits presented by Arita el al. (1). In the low beta-diversity graph, the minimum average covariance determines the lower boundary of the permitted region. In the high beta diversity region the lower bound is normally too near to the abscises and to the vertical line as to be displayed. However, in the high beta graph there is another curved line to the left of the vertical line, obtained by plotting (2) with the minimum negative value of the average covariances for the corresponding dataset.

Finally, the area of the total mathematically permitted region in a diversity-range plot can be obtained by simple integration and is$\left(\bar{\tau}_{\max}-\bar{\tau}_{\min}\right)\ln\left(\alpha_{\max}-\alpha_{\min}\right)$.
