# Supplementary figures and images for: Species Richness and Range Size of the Terrestrial Mammals of the World: Biological Signal within Mathematical Constraints
Source: PLoS One. 2011 May 6;6(5):e19359. doi: 10.1371/journal.pone.0019359 (PMC3089617; doi:10.1371/journal.pone.0019359)

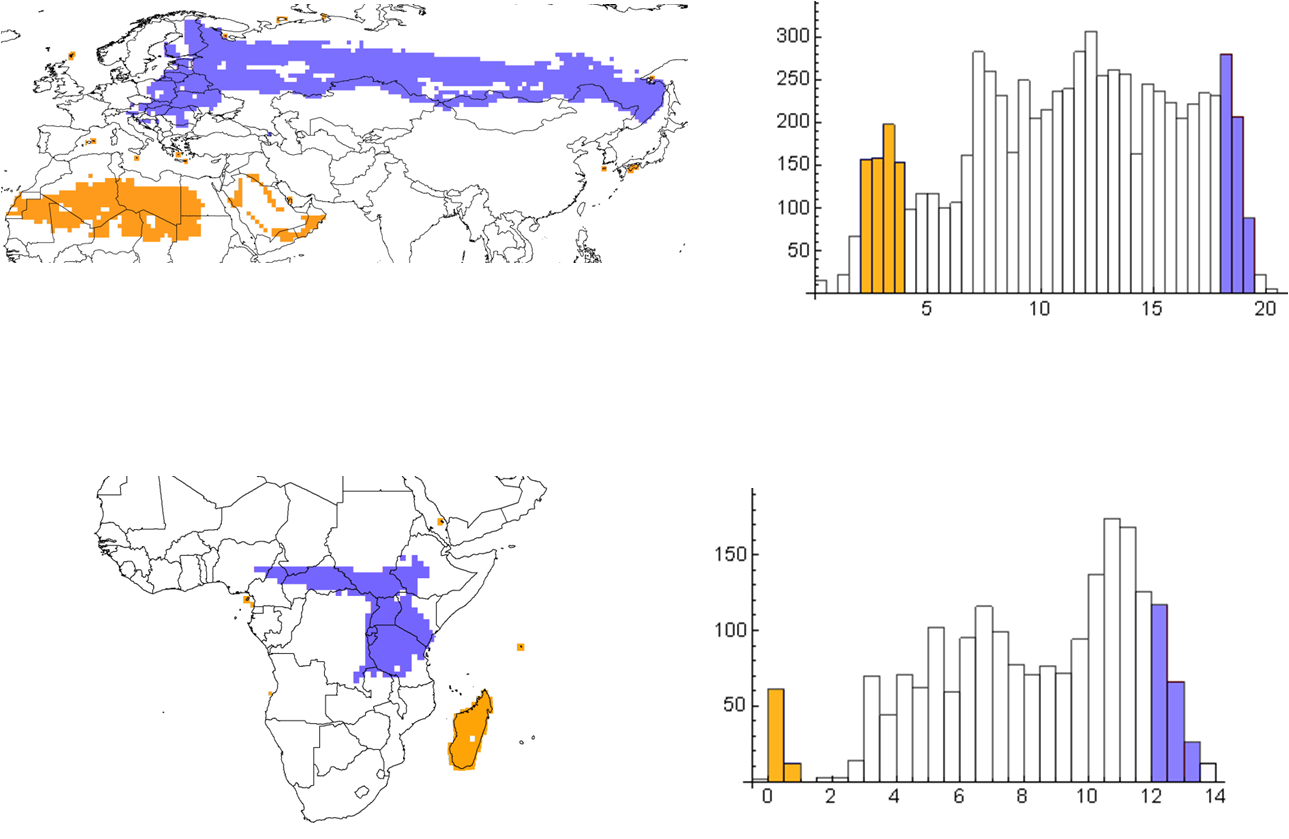

Supplement: Figure S1 — Extreme covariances and corresponding geographic regions for the Palearctic and Afro Tropical Regions. Histogram of covariances ×104 (right column) and corresponding regions in the map, displaying the fact that extreme values of covariance are associated to large, contiguous regions of similar ecological conditions (high covariance, purple) or to islands or peripheral regions of regions (low covariance, orange). (TIF) [file pone.0019359.s004.tif]

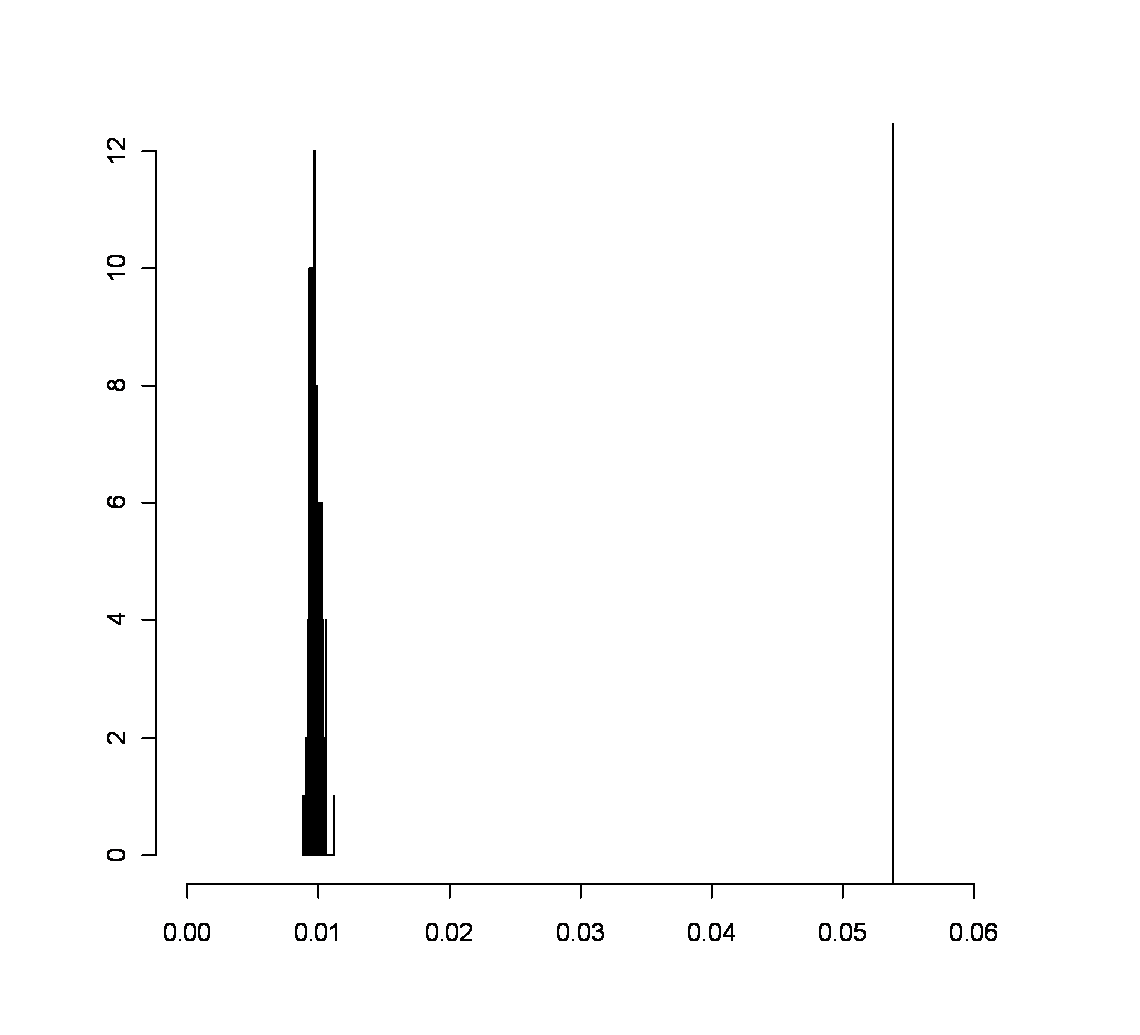

Supplement: Figure S2 — A histogram of the range of values for the range in mean covariance in 100 EcoSim randomizations (A), and in the observed Nearctic PAM (B). (TIF) [file pone.0019359.s005.tif]
